# 

**Published:** 1910-03-19

**Authors:** 


### GRADUATES' MEDICAL SCHOOLS.

DIARY FOR THE WEEK, MARCH 21 to MARCH 26.

### MEDICAL GRADUATES' COLLEGE AND POLYCLINIC, 22 Chenies Street, W.C.

At 4 p.m.—**March 21**, Dr. Wilfred Fox, **Clinique (skin).**
5.15 p.m.—**March 21**, Mr. W. Arbuthnot Lane, **Intestinal Stasis.**
4 p.m. — **March 22**, Dr. Guthrie Rankin, **Clinique (medical).**
5.15 p.m.—**March 22**, Dr. Norman Walker (Edinburgh), **The Detective Spirit in Dermatology.**
4 p.m.—**March 23**, Mr. P. J. Freyer, **Clinique (surgical).**
5.15 p.m.—**March 23**, Dr. J. M. H. MacLeod, **The Treatment of Nævi and Moles.**
4 p.m — **March 24**, Mr. Jackson Clarke, **Clinique (surgical).**
5.15 p.m. — **March 24**, Dr. G. E. Herman, **Placenta Prævia.**

### LONDON SCHOOL OF CLINICAL MEDICINE, Seamen's Hospital, Greenwich, S.E.

At 3.15 p m.—**March 21**, Sir Dyce Duckworth, **Diagnosis of Some Forms of Hepatic Enlargement.**
3.15.—**March 24**, Sir Wm. Bennett,

### NORTH-EAST LONDON POST-GRADUATE COLLEGE, Prince of Wales's General Hospital, Tottenham, N.

At 4.30 p.m.—**March 23**, Mr. R. P. Brooks, **Selected Eye Cases.**

### THE BROMPTON HOSPITAL FOR CONSUMPTION AND DISEASES OF THE CHEST, Fulham Road, S.W.

At 4 p.m.—**March 23**, Dr. Inman, **Laboratory Aids to Diagnosis and Treatment.**

### CENTRAL LONDON THROAT AND EAR HOSPITAL, Gray's Inn Road, W.C.

At 3.45 p.m.—**March 22**, Mr. J. Gay French, **Tracheoscopy, &c.**

3.45 p.m.—**April 1**, Mr. W. Hoden George, **Anæsthetics.**

### THE POST-GRADUATE COLLEGE, West London Hospital, Hammersmith, W.

At 10 a.m.—**March 21 and 24, Demonstration by Surgical Registrar.**
10 a.m.—**March 22, Demonstration by Medical Registrar.**
12 noon.—**March 24**, Dr. Bernstein, **Pathological Demonstration.**
12.15 p.m.—Dr. Grainger Stewart, **Practical Medicine.**
5 p.m.—Dr. Beddard, **Medical Cases.**
5 p.m.—Mr. Pardoe, **Clinical Lecture.**
5 p.m.—Mr. Dunn, **Tobacco Amblyopia.**

Congress of Physiotherapy (Special Tickets).—The following special reduced rates for the members or associates and their families who will attend the Third International Congress and Exhibition of Physiotherapy (Paris, March 29 to April 3, 1910) have been obtained. The South Eastern and Chatham Railway will issue their tickets from March 25 until April 2 at Charing Cross booking office, by the short sea and mail routes, *via* Dover and Calais, and *via* Folkestone and Boulogne, at : 58s. 4d., first class return fare to Paris, instead of 95s. 9d.; 37s. 6d., second class return fare to Paris, instead of 69s. 10d. The London, Brighton and South Coast Railway will issue their tickets at Victoria or London Bridge stations, *via* Newhaven and Dieppe, at : 39s. 3d., first class return fare to Paris, instead of 66s. 3d.; 30s. 6d., second class return fare to Paris, instead of 47s. 1d. The tickets will be available by day or night services, and will have a validity of one month. These privileged tickets will be delivered by the two companies only on presentation of the members' or associates' admission cards. Should several members or associates wish to travel together in groups, M. Saunion will make special arrangements for the comfort of the travellers, throughout to Paris, not only on the English railways but also on the French ones.

London School of Clinical Medicine.—The annual dinner of the London School of Clinical Medicine (Post Graduate) was held on Tuesday night last at the Hotel Cecil. Sir James Crichton-Browne presided, and there was a good attendance, the guests including Sir Archibald Geikie, Surgeon-General Gubbins, Admiral Sir John Durnford, Sir Frederick Bridge, and Inspector-General Porter. The Chairman, after the usual loyal toasts had been honoured, proposed the toast of the evening, "The London School of Clinical Medicine," and said that although the school was prospering and out of debt, there were several things which the executive desired to obtain in order to improve the usefulness of the school. Thus a pathological laboratory in charge of a paid pathologist was urgently desirable, and he looked to some Rockefeller or other good friend of the institution to give the Secretary or Dean a cheque for five thousand pounds, in order to enable the pathological laboratory to be started. The health of the guests was responded to by Sir Archibald Geikie, who referred to the usefulness of post-graduate study, and gave a short account of what the Royal Society has done in investigating sleeping sickness, by Inspector-General Porter, who paid a high tribute to the school on behalf of the Navy, by Surgeon-General Gubbins, who responded on behalf of the Army, and by Dr. Ogilvie, who returned thanks for the other guests.

### Appointments.

Dr. A. E. L. Wear has been appointed school medical officer of Leeds.

Dr. H. C. T. Langdon has been appointed medical officer of health of Mansfield.

Dr. M. D. Ferguson has been appointed house physician in place of Dr. Stanley Upton, resigned, at York County Hospital.

Dr. Grahame H. Skinner, of Stirling, and Dr. Josephine Gardner, of Ilkeston, have been appointed medical officers of health for the county of Stirlingshire.

Mr. T. Miller Neatby, M.A., M.D. Cantab., and M.A. London, and Mr. John Weir, M.B. Glasgow, have been appointed assistant-physicians to the Homœopathic Hospital.

Mr. Edward Daniell Parsons, M.R.C.S., L.R.C.P., D.P.H., assistant medical officer of health, Croydon, has been appointed assistant medical officer of health of Northampton.

Three important appointments have recently been made to the medical staff of the London Homœopathic Hospital: Mr. Giles F. Goldsbrough, M.D. Aberdeen, having been appointed visiting physician in place of Mr. Byres Moir, M.D. Edinburgh, who has retired and been appointed a consulting physician to the hospital.

### Gifts and Bequests.

An anonymous lady has sent 250 guineas to the London Fever Hospital.

Mrs. Ada Britannia Sarah Peters, of Kilburn, £100 to the Anti-Vivisection Hospital, Brompton.

The Hospital and Home for Incurable Children, Hampstead, has received £250 from Mr. R. Nivison.

Miss Elizabeth Mary Gell, of Douglas, Isle of Man, has left £500 to the Noble Isle of Man Hospital.

Dr. William Thomas Bensly, F.S.A., solicitor, Registrar of the Diocese of Norwich, has left £50 to the Norfolk and Norwich Hospital.

Lady Lusk, of Hyde Park, W., has left £1,000 to St. Mary's Hospital for the endowment of a bed to be known as the "Lusk" bed.

Mr. John Edward Ward, of Shotter Mill, Surrey, has left £50 to the Westminster Hospital and £50 to the Royal Asylum of St. Anne's Society.

The Earl Cawdor, Treasurer of the London Homœopathic Hospital, Great Ormond Street, W.C., has received £100 from Mr. Otto Beit, being his annual subscription to the funds of the institution, and a cheque for £4,500, being the legacy of £5,000 bequeathed by the late Mrs. Evans, of Roehampton, less £500 legacy duty.

Mr. Robert Harrison, of Lincolnshire, farmer, has left £500 each to the Horncastle Dispensary and the Lincoln County Hospital, and real estate at Martin Dales and Walcot Fen, Lincs, of which he was mortgagee in possession, upon trust for sale and to pay the proceeds in equal shares to the Lincoln County Hospital and the Horncastle Dispensary.

Miss Adela Fanny Barlow, of Dover, has left £300 each to the Chelsea Hospital for Women, the National Hospital for diseases of the Heart, the Evelina Hospital for Sick Children, the Royal Ear Hospital, Soho, the Cancer Hospital, Fulham Road, the cancer wing of Middlesex Hospital, the National Free Home for the Dying, and the Royal Sea Bathing Hospital, Margate.

### THE QUESTION BOX.

**SPECIAL NOTICE.**—Questions of interest affecting the honorary medical staff and hospital administration in all departments are invited. When any point arises we hope our readers will formulate it in a question and so co-operate to make this column of real value and interest. In this way the experience of the best-managed hospitals should be made helpful to the whole hospital world. We shall welcome the contribution of both questions and answers.

**N.B.**—The publication of an answer in this column does not necessarily preclude the publication of subsequent answers to the same question, for a question may involve points which require to be threshed out and fully discussed. Answers, if published, will be paid for at scale rates.

### MEDICAL AND SURGICAL STATISTICS IN HOSPITAL REPORTS.

Question: Some attention has been directed to the question of these returns and their relative value or worthlessness. Has any hospital at present made an attempt to remodel these statistics and to make them represent something definite and prove of scientific value, which each governor and the public generally may grasp and understand?

[Replies to this question are invited.]

### A CENTRAL STORE FOR HOSPITALS.

We defer publishing further contributions on this subject to enable us to give a summary of an interesting paper on "The Hospital Drug Bill," which is written with great ability and shows an insight into the business which confers the greatest credit upon its author. As we mentioned last week, there is some prospect of an experiment being made in co-operative buying by a group of hospitals in East London, with the London Hospital at their head, which, if found practicable, will afford some useful experience of the possibilities of such a system. We incline to think that in this connection it would be found of value to take advantage of the offer of the managing director of the great stores, whose views we published last week. Such a step would obviate several of the difficulties which Mr. Hocking has clearly enunciated and driven home. It would remove the necessity of providing any initial capital, and would practically give the combining hospitals, without cost to themselves, all the advantages which the central warehouse scheme referred to below might provide. Two practical facts, however, must always be borne in mind in this connection: (1) that the possible saving is not likely to exceed 10 and will probably only amount to 5 per cent., and (2) that the cost of delivering all the supplies to the co-operative hospitals may in practice more than absorb the whole of this saving.

### THE HOSPITAL DRUG BILL.

Mr. Hocking, B.Sc., has written a very practical and informing paper on this subject which appears in the current number of the *Hospital Gazette*. Mr. Hocking's great practical experience, and the fulness of practical knowledge which his paper exhibits, give his utterances unusual point and force. He challenges the claim that the manufacture of galenicals by hospitals necessarily means superior quality or the "tremendous saving" likely to result to a hospital which actually makes its pills and galenicals. He supports his scepticism by clear and forcible argument. He further shows by quoting instances the wide difference in the prices paid for apparently the same articles by different institutions, though he omits to emphasise the importance of uniformity of product and quality in the articles dealt with, as, failing these essentials, difference in price is easily accounted for. He maintains that the popular idea that purchases in large quantities mean a reduction in the price is not necessarily well founded, and proceeds to show by examples that the probable difference in price would average something like 5 per cent. only. Having made his various points clearly, Mr. Hocking suggests for consideration the abolition of all the galenical laboratories for both large and small hospitals, and the establishment in their place of one great central warehouse, laboratory, buying centre, and distributing agency for the whole of the Metropolitan voluntary hospitals. That is to say, he suggests the creation of a wholesale house owned and run by the hospitals for their exclusive use and benefit.

Basing his estimates on the supposition that St. Bartholomew's and all the institutions sending returns to the King's Fund entered such a scheme, there would then be sixty-seven hospital co-operating, with a total annual expenditure under the heading of surgery and dispensing in 1908 of upwards of £93,000. After making certain necessary deductions for instruments and so forth, he puts the approximate annual turnover for such a central warehouse at £70,800. He then gives a list of the staff required to work such a warehouse and its bearing upon the position of existing hospital officers in the departments affected; and mentions the possibility of a combination of the existing wholesale houses, with a view to boycotting any such attempt, and its probable success or failure. Mr. Hocking estimates the capital required to work such a scheme as this at upwards of £20,000, and wonders if one of the central funds would consent to employ some of their reserves at 4 per cent. interest by investing them in such a central warehouse on the understanding that an additional 1 per cent. was provided out of the profits of such a venture as a sinking fund for the redemption of this capital. As the security offered is not a trustees' security but a commercial speculation, the trust funds mentioned by Mr. Hocking would not seem available for the purposes mentioned. Mr. Hocking has contributed a most interesting and practical paper of the first importance, and the conclusions which will probably result from it are that the advantages which might possibly accrue from co-operation are likely to be largely discounted by the expenses and difficulties of working such a scheme.

---

A meeting of medical men in favour of the general principles laid down in the Minority Report of the Poor Law Commission was held on Tuesday last at the rooms of the Royal Society of Medicine. Dr. Lawson Dodd presided, and Mr. Sidney Webb briefly explained the outline of the scheme advocated in the Minority Report. A provisional committee was appointed, with Dr. Becket Overy and Mr. Somerville Hastings as hon. secretaries, to consider further action in the matter.

THE HOSPITAL. March, 19, 1910.

*Name* ........................................

*Address* ........................................

........................................

This Coupon must accompany manuscript or contributions intended for The Hospital.

